# Fellow-Workers and Their Doings

**Published:** 1914-04-11

**Authors:** 


### ROUND THE WORLD.

### Fellow-Workers and their Doings.

**[The Editor Invites Early Information and Contributions Marked "Fellow-Workers."]**

A preliminary announcement has been made, to be followed later by a further list, of special researches authorised by the President of the Local Government Board and paid for out of the annual grant at his disposal for the investigation of disease. Dr. Eardley Holland will review the causes of still-birth. He is physician to the City of London Lying-in Hospital and assistant obstetric physician to King's College Hospital, where his medical education was conducted. He has also held posts at Queen Charlotte's Hospital, the Hospital for Women, Soho, and Paddington Green Children's Hospital.

Professor Sims Woodhead will continue his inquiry into the cellular contents of milk. The Cambridge professor is of course an Edinburgh graduate, and was Director of Research in the laboratories of the Conjoint Board of the English Royal Colleges before he went to Cambridge about the end of last century. He is an honorary graduate of Toronto and Birmingham Universities, a member of the Executive Committee of the Imperial Cancer Research Fund, and an energetic advocate of total abstinence from alcohol.

The causes of premature arterial degeneration are to be further investigated by Dr. F. W. Andrewes. An Oxford and St. Bartholomew's man, he was formerly assistant physician to the Royal Free Hospital before devoting himself entirely to pathology. He is now sanitary officer, pathologist, and lecturer on pathology to St. Bartholomew's, and has been a prolific writer on bacteriological and pathological subjects. Dr. Andrewes has worked under the Local Government Board before, having been the author of articles in the Reports of that body in 1907, 1908, and 1910.

The ætiology of epidemic infantile diarrhœa is allotted to Drs. M. H. Gordon and A. E. Gow for investigation. They also are "Bart.'s" men; the former is assistant pathologist, the latter is medical registrar. Dr. Gordon has contributed to the Local Government Board Reports on numerous occasions in the last eleven years, and has also worked at the ventilation of the House of Commons. Dr. Gow has had experience at the Queen's Hospital for Children which should well qualify him for the clinical side of this joint research.

The Jacksonian prize of the Royal College of Surgeons has been awarded to Mr. J. H. Evans, F.R.C.S. The subject set for the competition was malformations of the small intestine. Mr. Evans is surgeon to the Prince of Wales's Hospital, Tottenham, and assistant surgeon to the Cancer Hospital, Fulham Road. He was educated at Oxford and at St. George's Hospital; at the latter, and also at the Chelsea Hospital for Women, he has been surgical registrar. Mr. Evans takes a special interest in mal-developments of all sorts, and has contributed several times to the literature of this subject.

A lecture recently given by Dr. A. C. Jordan, M.D., M.R.C.P., at the Royal Hospital for Diseases of the Chest, City Road, drew attention to some important points in the use of *x*-rays as an aid to the diagnosis of lung diseases. Discussing various co-called "signs" revealed by the *x*-rays when pulmonary tuberculosis is present, this investigator stated that the failure of the apex of a lung to become lighter when a deep breath is taken and the chest viewed through the radiographic screen is by no means as certain an indication of tubercular lesion as has been hitherto supposed. All practitioners who have much to do with consumptive cases may profit by Dr. Jordan's important dictum that the only sure *x*-ray sign in the detection of lung tubercle is "mottling." Since his retirement from St. Bartholomew's Hospital Dr. Jordan has carried out a great deal of work as medical officer in charge of the Rontgen-ray department at the Royal Chest Hospital.

Mr. Bilton Pollard, who has just retired from the staff of University College Hospital, to which he has been attached for many years, served there in the various grades of the surgical staff, and also as Professor of Clinical Surgery at University College. He qualified M.R.C.S. in 1879, taking the "Fellowship" two years later; in addition to which diplomas he holds the double degree of London University. Mr. Pollard has for some time enjoyed a prominent place in the front rank of London consultants, and is well known to students not only through his writings, but by his former posts of examiner in surgery to the University of Oxford and the Victoria University, as well as in anatomy at the Royal College of Surgeons. It is understood that in future Mr. Pollard intends to spend a good deal of time at his place in Sidmouth. Of late years he has actively followed the important developments in abdominal surgery.

